# Establishment of Reference Interval and Aging Model of Homocysteine Using Real-World Data

**DOI:** 10.3389/fcvm.2022.846685

**Published:** 2022-03-30

**Authors:** Chaochao Ma, Lei Li, Xinlu Wang, Li’an Hou, Liangyu Xia, Yicong Yin, Xinqi Cheng, Ling Qiu

**Affiliations:** ^1^Department of Laboratory Medicine, Peking Union Medical College Hospital, Peking Union Medical College and Chinese Academy of Medical Sciences, Beijing, China; ^2^Department of Medical Laboratory Technology, Public Health College, Nanchang University, Nanchang, China; ^3^State Key Laboratory of Complex Severe and Rare Diseases, Peking Union Medical College Hospital, Peking Union Medical College and Chinese Academy of Medical Sciences, Beijing, China

**Keywords:** aging model, homocysteine, reference interval, big data mining, GAMLSS algorithm

## Abstract

**Objective:**

The level of Homocysteine (Hcy) in males is generally higher than that of females, but the same reference interval (RI) is often used in clinical practice. This study aims to establish a sex-specific RI of Hcy using five data mining algorithms and compare these results. Furthermore, age-related continuous RI was established in order to show the relationship between Hcy concentration distribution and age.

**Methods:**

A total of 20,801 individuals were included in the study and Tukey method was used to identify outliers in subgroups by sex and age. Multiple linear regression and standard deviation ratio (SDR) was used to determine whether the RI for Hcy needs to be divided by sex and age. Five algorithms including Hoffmann, Bhattacharya, expectation maximization (EM), kosmic and refineR were utilized to establish the RI of Hcy. Generalized Additive Models for Location Scale and Shape (GAMLSS) algorithm was used to determine the aging model of Hcy and calculate the age-related continuous RI.

**Results:**

RI of Hcy needed to be partitioned by sex (SDR = 0.735 > 0.375). RIs established by Hoffmann, Bhattacharya, EM (for females) and kosmic are all within the 95% CI of reference limits established by refine R. The Sex-specific aging model of Hcy showed that the upper limits of the RI of Hcy declined with age beginning at age of 18 and began to rise approximately after age of 40 for females and increased with age for males.

**Conclusion:**

The RI of Hcy needs to be partitioned by sex. The RIs established by the five data mining algorithms showed good consistency. The dynamic sex and age-specific model of Hcy showed the pattern of Hcy concentration with age and provide more personalized tools for clinical decisions.

## Introduction

Homocysteine (Hcy) is a sulfur-containing amino acid that is derived in methionine (Met) metabolism (1). Hcy has been recognized as an independent risk factor for cardiovascular diseases, since 1969 McCully identified the elevated Hcy level was linked to premature vascular disease (2). Today, it is widely accepted that whenever there is an elevation of Hcy in the body, whether genetic or acquired, that leads to the generation of numerous diseases, such as cardiovascular diseases (3). Therefore, Hcy level should be monitored in regular medical examination for both the risk assessment considering the association between homocysteine and cardiovascular disease. However, the reference interval (RI) used in clinical practice is mostly provided by reagent manufacturers’ instructions, and the RI of Hcy is not partitioned according to sex. Because males have higher Hcy levels than females (4), the use of only one RI may misestimate the proportion of abnormally elevated Hcy in males and normal proportion in females. Therefore, it is particularly important to establish a sex-specific RI for Hcy.

In present, methods for establishing RIs are divided into direct sampling method and indirect sampling method (5, 6). Direct sampling method is currently the preferred method for establishing RIs. However, this method has high cost and complicated steps, and not all clinical laboratories have the condition to establish RIs using direct sampling method. In recent years, with the development of the computer and the popularity of data mining, using real-world big data to establish RIs, also known as indirect sampling method, has attracted more and more attention (6). This method does not require baseline information about subjects and uses data from individuals undergoing routine medical examinations at the hospital. After the outliers are removed, the distribution of healthy subjects can be separated from a mix of data including non-pathological (physiological) and pathological test results using an algorithm similar to unsupervised clustering. The process is simple, low cost and feasible, which is a great supplement to the direct sampling method. Currently, the main indirect sampling methods include Hoffmann method (7), Bhattacharya method (8), Expectation Maximization (EM) algorithm (9), kosmic algorithm (10), refineR algorithm (11) and so on. These algorithms are based on different principles to separate the mixed distribution and obtain the data distribution parameters of healthy individuals, each of which has advantages and disadvantages. Based on this, the study will use the big data of the population who underwent a physical examination to analyze whether RI of Hcy need to be partitioned by sex and age using the variance component model. In addition, Hoffmann, Bhattacharya, EM, kosmic and refineR algorithm will be used to establish the RI of Hcy, and the consistency of the RI of Hcy established by the five algorithms is about be compared. The last but not least, considering that previous study has reported that the mean Hcy concentration of the population changed with age and the relationship between them is not linear (12), this study will adopt generalized additive models for location, scale and shape (GAMLSS) algorithm to fit the model of Hcy level and age, and to establish the age-related continuous RI for Hcy by using the aging model. The results of this study will not only provide a more personalized tool for clinical decision-making, but also provide practical experience and theoretical reference for clinical laboratory to establish the RI.

## Materials and Methods

### Study Design and Data Cleaning

A total of 20,801 individuals who underwent a medical examination and tested Hcy in Peking Union Medical College Hospital from May 2020, to December 2021 were included in this study. This study mainly includes two steps which listed as following.

Step 1: Data cleaning

(1) Check to see if there are missing values of age, sex and Hcy in the data.
(2) Determine whether the Hcy results of all enrolled individuals were analyzed using the same assay platform.
(3) Sex was transformed into a factor variable, with female marked 0 and male marked 1.
(4) Age was grouped at 18–29 years, 30–39 years, 40–49 years, 50–59 years, 60–69 years, 70–79 years and > 80 years.
(5) Subjects aged 18 years or older was included in the study (20,801–20,796).
(6) Limit results to a single result per subject. In other words, if subjects have had multiple medical examination results during the study period, retain their initial results (20,796–17,640).
(7) Individuals with less folate than the reference limits (< 4.0 ng/mL) (17,640–17,318).
(8) Results of vitamin B12 not within the reference interval (< 180 pg/mL or > 914 pg/mL) were excluded (17,318–16,022).
(9) Results of alanine aminotransferase (ALT) not within the reference interval (female: 7–40, Male: 9–50 U/L) were excluded (16,022–14,908).
(10) Results of aspartate amino transferase (AST) not within the reference interval (Female: 13–35, Male:15–40 U/L) were excluded (14,908–13,945).
(11) Results of creatinine (Cr) not within the reference interval (Female: 45–84, Male:59–104 μmol/L) were excluded (13,945–13,527).
(12) Results of urea not within the reference interval (2.78–7.14 mmol/L) were excluded (13,527–12,687).
(13) Results of thyroid stimulating hormone (TSH) not within the reference interval (0.380–4.340 μIU/L) were excluded (12,687–11,507).
(14) Results of free triiodothyronine (FT3) not within the reference interval (1.80–4.10 pg/mL) were excluded (11,507–11,334).
(15) Results of free thyroxine (FT4) not within the reference interval (0.81–1.89 ng/dL) were excluded (11,334–11,311).
(16) In order to ensure that the RI of Hcy would not be deviated due to the difference in the number of females and males or the difference in the number of individuals in different age groups, a new data set named data set 1 was formed by randomly sampling from each subgroup with equal-weighted and removed outliers using Tukey method in each subgroups. The sample size of data set 1 is 2,261.
(17) After grouping 11,311 individuals according to sex, Tukey method was used to check and remove outliers, and this operation was repeated until no value was removed as outliers. Then, construct data set 2 utilizing 11,074 individuals’ data without outliers.

Step 2: Data Analysis

(1) Based on data set 1, the influences of sex and age on Hcy level were analyzed and the RIs of Hcy were established using five data mining algorithms including Hoffmann, Bhattacharya, EM, kosmic and refine R.
(2) Based on data set 2, aging model of Hcy was established using Generalized Additive Models for Location Scale and Shape (GAMLSS) algorithm and validated by using the randomly split internal validation set (see Statistical Analysis for detailed description).

### Ethical Approval

This retrospective study was part of the big data project (CFH-2020-1-4014) and was approved by the Ethics Committee of Peking Union Medical College and Chinese Academy of Medical Sciences, Peking Union Medical College Hospital (approval number: S-K1192).

### Analytical Performance of Analytes and Quality Control

Concentration of Hcy was measured on the AU5800 automatic biochemical analyzer (Beckman Coulter, Brea, CA, United States), using reagents and calibrators supplied by the LEADMAN (Beijing, China). Intra-assay and inter-assay precision provided by the specification of the manufacturer was less than 8% and 15%. The total coefficient of variation for low and high levels of Hcy calculated from the results of daily indoor quality control was 3.28 and 4.28% in our clinical laboratory, respectively. The detection range is 2.5–50μmol/L. If the test results are more than the linear range, dilute with sterile water or normal saline. The RI of Hcy in serum provided by the manufacturer was 15 μmol/L. Coagulation-promoting tubes (Vacuette, Greiner Bio-One GmbH, Frickenhausen, Germany) were utilized to collect the samples in order to reduce the effect of serum on levels of Hcy. Once serum was collected. The samples were tested immediately. Hcy participated in external quality assessments conducted by the National Center for Clinical Laboratories and by the College of American Pathologists two times a year to ensure that our test results are correct and reliable. The assay platform for the analyte did not change during the time period covered by the data. In addition, quality control (QC) checks were also conducted twice daily on Hcy. Specimen measurement was conducted only after qualifying quality control.

### Statistical Analysis

All data were recorded in 2016 Microsoft Excel sheet (Microsoft, Redmond, WA, United States), then analyzed using packages (10, 11) and codes (13) implemented in R language (version 4.0.5), as well as SPSS 25.0 Software (IBM Inc., Armonk, NY, United States). Tukey method was used to test outliers in subgroups considering differences in subgroups. The Box-Cox transformation was conducted to normalize the data before using the Tukey method. Tukey method involves the computation of the 25th (Q1) and 75th (Q3) percentiles and the interquartile range (IQR = Q3 − Q1). Values were excluded if they less than Q1 − 1.5*IQR or more than Q3 + 1.5*IQR. A maximum likelihood algorithm was implemented to calculate the lambda value. Multiple linear regression was carried out to identify effects of sex and age on levels of Hcy. Considering the relationship between concentration of Hcy and age are not a straight-line, age was set as dummy variables. In multiple linear regression, female and 18–29 years were, respectively, set as reference level in sex and age. Standardization regression coefficient (β) were used to evaluate the effects of sex and age on the concentration of Hcy and identify the hierarchy of sex and age in the variance components model. Due to the large sample size, the reference value of *p*-value in multiple linear regression is insignificant. Therefore, we utilized variance components model to calculate standard deviation and variance of age and sex. Furthermore, standard deviation ratio (SDR) was expressed as (SDsex or SDage)/SDresidual and was employed to judge whether RI of Hcy needs to be divided into several partitioning by sex and age. 0.375 is used as a judgment threshold. Five data mining algorithms including Hoffmann, Bhattacharya, EM, kosmic and refineR were used to establish 95% right-side RIs of Hcy. And 95% confidence interval (CI) of RI was also calculated and used to judge whether the reference limits established by five algorithms were different, that is, reference limits are within 95% CI shows that there is no difference. GAMLSS model was used to show the relationship of concentration of Hcy and age. More importantly, RIs of Hcy could be established by GAMLSS model according to unit of 1 year. In establishment of GAMLSS model for Hcy and age, the data set 2 was randomly split into a training data set (70% of the data) and a validation data set (30% of the data). Training data set was used to establish models and validation data set was used to calculate the percentage of individuals who fall outside the reference interval (FOR) according to unit of 1 year. The mean of FOR value should be less than 10% and around 5%. Generalized Akaike Information criterion was used to assess goodness of fit and choose model. Levels of significance (α) were set at 0.05.

## Results

### Information of Data Sets 1 and 2

The basic information is shown in the Table 1. The sample size of data set 1and data set 2 is 2,261 and 11,074. The sex ratio of data set 1 is 1:1, while data set 2 consists of 7,685 females and 3,385 males. The age distribution of data set 1 and 2 is 50 (36, 63) and 47 (38, 55), respectively. The folate level of individuals in the data set 1 and 2 are 10.4 (7.8, 14.8) and 10.9 (8.2, 14.9) ng/mL. The vitamin B12 level of individuals in the data set 1 and 2 are 356 (281, 475) and 368 (288, 479) pg/mL, respectively. Subjects enrolled in the study has normal ALT, AST, Cr, Urea, TSH, FT3 and FT4.

**TABLE 1:** The basic information of data set 1 and data set 2.

	Units	Data set 1	Data set 2
*n*		2,261	11,074
Sex	Female: male	1,173:1,124	7,685:3,385
Age	Year	50 (36, 63)	47 (38, 55)
Vitamin B12	pg/mL	356 (281, 475)	368 (288, 479)
Folate	ng/mL	10.4 (7.8, 14.8)	10.9 (8.2, 14.9)
ALT	U/L	18 (14, 25)	16 (12, 23)
AST	U/L	20 (17, 23)	19 (16, 22)
Cr	μmol/L	70 (61, 80)	65 (58, 75)
Urea	mmol/L	4.94 (4.28, 5.66)	4.77 (4.09, 5.53)
TSH	μIU/L	1.903 (1.381, 2.602)	1.888 (1.366, 2.589)
FT3	pg/mL	3.32 (3.11, 3.55)	3.27 (3.06, 3.48)
FT4	ng/dL	1.23 (1.14, 1.34)	1.22 (1.12, 1.32)

*Results were description as Median (P25, P75).*

### Effects of Sex and Age on Concentration of Homocysteine

**FIGURE 1:**
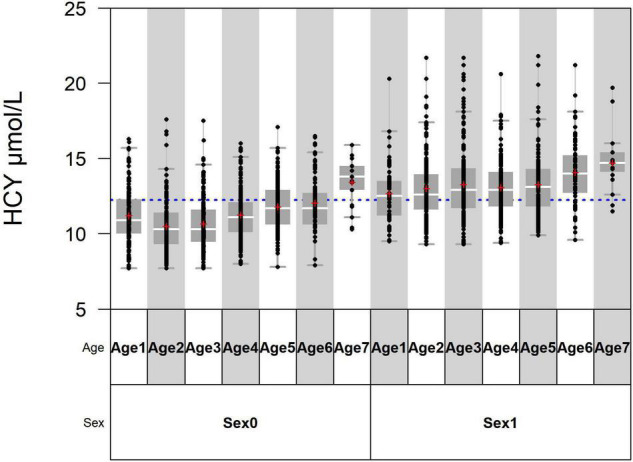
Age 1, Age 2, Age 3, Age 4, Age 5, Age 6, and Age 7 are, respectively, standing for 18–29 years, 30–39 years, 40–49 years, 50–59 years, 60–69 years, 70–79 years, and > 80 years. Sex0 stands for females and sex1 stands for males.

The standardized regression coefficient of sex is 0.448, which is larger than that of age (Table 2). In the variance component model, sex is placed on the first level and age is placed on the second level (Figure 1). The average level of Hcy in males of different age groups was greater than in females (*P* < 0.05). SDR of sex is 0.735, greater than 0.375, while SDR of age is 0.312, less than 0.375 (Table 3). This suggests RI of Hcy needs to be partitioned by sex.

**TABLE 2:** Results of multiple linear regression.

	Sex		Age											
			A1		A2		A3		A4		A5		A6	
							
	β	*P*	β	*P*	β	*P*	β	*P*	β	*P*	β	*P*	β	*P*
**HCY**	0.448	< 0.001	−0.033	0.160	0.004	0.874	0.038	0.110	0.101	< 0.001	0.179	< 0.001	0.146	< 0.001

***β**, Standardized regression coefficient. The standardization regression coefficient (**β**) was used to measure the weights of influential factors and identify the hierarchy of sex and age in variance components model; Female is the reference for sex groups. Age was transformed into six dummy variables (A1, A2, A3, A4, A5, A6): (18–29 = 0,0,0,0,0,0; 30–39 = 1,0,0,0,0,0; 40–49 = 0,1,0,0,0,0; 50–59 = 0,0,1,0,0,0; 60–69 = 0,0,0,1,0,0; 70–79 = 0,0,0,0,1,0; 80 + = 0,0,0,0,0,1). 18–29 year is the reference level. A1 stands for 30∼39 years relative to 18∼29 years, A2 stands for 40∼49 years relative to 18∼29 years, A3 stands for 50∼59 years relative to 18∼29 years, A4 stands for 60∼69 years relative to 18∼29 years, A5 stands for 70∼79 years relative to 18∼29 years, A6 stands for age 80 or older relative to 19∼29 years.*

**TABLE 3:** SDR of sex and age.

	SDresi	Sex		Age	
		*SD*	SDR	*SD*	SDR
**HCY**	1.876	1.378	0.735	0.586	0.312

*SD, standard deviation; SDR, standard deviation ratio.*

### Reference Interval of Homocysteine by Using Five Algorithms

**FIGURE 2:**
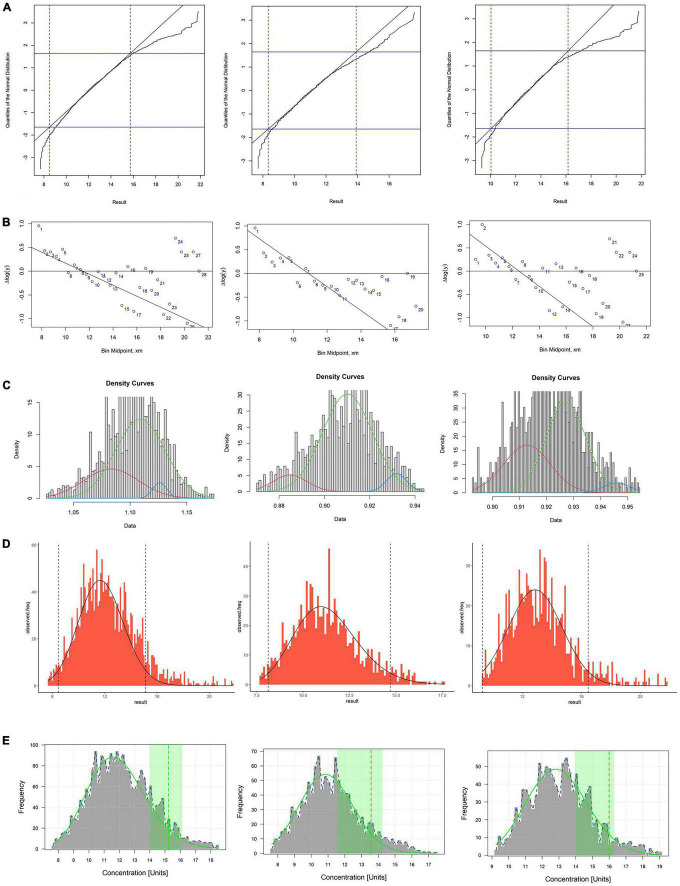
**(A–E)** Respectively, stand for Hoffman, Bhattacharya, EM, kosmic and refineR algorithms. The first column represents the total reference interval, the second column represents the reference interval for females, and the third column represents the reference interval for males. **(A,B)** In the plot of Hoffmann and Bhattacharya, linear region represents the distribution of healthy individuals and RI were determined by extending the linear region of the healthy subgroup. **(C)** The green curve represents the distribution of healthy individuals and RI were determined by distribution of green curve. **(D)** The algorithm minimizes the difference between an estimated parametrical distribution and a truncated part (the interval between two vertical dashed lines) of the observed distribution, and RI were determined by estimated parametrical distribution (the distribution curve in the graph). **(E)** The green curve in plot represents the distribution of healthy individuals and the green vertical dotted line indicates the upper limit of the estimated RI.

The RIs of Hcy established by using five data mining algorithms are shown in the Table 4 and Figure 2. The results of the five algorithms are consistent whether it is the total RI of Hcy or the sex-specific RI. The upper limits of RI established by Hoffmann, Bhattacharya, EM (for female) and kosmic are all within the 95% CI of reference limits established by refineR. The upper limit of RI established by five algorithms was higher in males than in females.

**TABLE 4:** Reference interval of Hcy established by using five algorithms.

		Hoffmann	Bhattacharya	Expectation maximization	kosmic	Refine R	
Total	P2.5	7.8	7.6	9.5	8.5	8.4	7.92–8.79
	P5	8.5	8.3	9.9	9.0	8.9	8.53–9.21
	P25	10.6	10.3	11.3	10.5	10.5	10.30–10.76
	Median	12.1	11.7	12.5	11.6	11.7	11.42–12.10
	P75	13.6	13.1	13.9	12.8	13.0	12.50–13.60
	**P95**	**15.7**	**15.1**	**16.5**	**14.5**	**15.0**	**14.00–16.13**
	P97.5	16.4	15.8	17.6	15.1	15.7	14.48–17.06
Female	P2.5	7.8	8.4	9.0	8.1	7.9	7.38–9.82
	P5	8.3	8.8	9.3	8.5	8.3	7.95–10.08
	P25	10.0	10.2	10.3	9.9	9.8	9.62–10.93
	Median	11.1	11.1	11.1	11.0	10.9	10.32–11.53
	P75	12.3	12.1	12.1	12.1	11.9	10.85–12.29
	**P95**	**14.0**	**13.5**	**13.9**	**14.0**	**13.6**	**11.56–14.24**
	P97.5	14.4	14.0	14.6	14.7	14.1	11.79–14.95
Male	P2.5	9.4	9.8	11.4	9.3	9.7	8.97–11.24
	P5	10.0	10.3	11.8	9.9	10.1	9.63–11.59
	P25	11.8	11.7	12.8	11.6	11.7	11.29–12.73
	Median	13.1	12.7	13.7	12.9	12.9	12.17–13.58
	P75	14.4	13.8	14.7	14.1	14.1	12.91–14.51
	**P95**	**16.2**	**15.2**	**16.4**	**15.9**	**15.8**	**13.97–16.30**
	P97.5	16.8	15.7	17.1	16.5	16.4	14.31–17.05

*The results of EM algorithm are estimated using mean and standard deviation; P2. 5, P5, P25, P75, P95, P97.5 represents 2.5 quantile, 5 quantile, 25 quantile, 75 quantile, 95 quantile, and 97.5 quantile, respectively; The P95 quantile is the upper limit of the reference interval established in our study and the lower limit is 0. Hoffmann, Bhattacharya, Expectation maximization, kosmic, Refine R represents five algorithms which were used in our study. The bold values are the upper limits of RI for Hcy.*

### Sex-Specific Aging Model of Homocysteine

**FIGURE 3:**
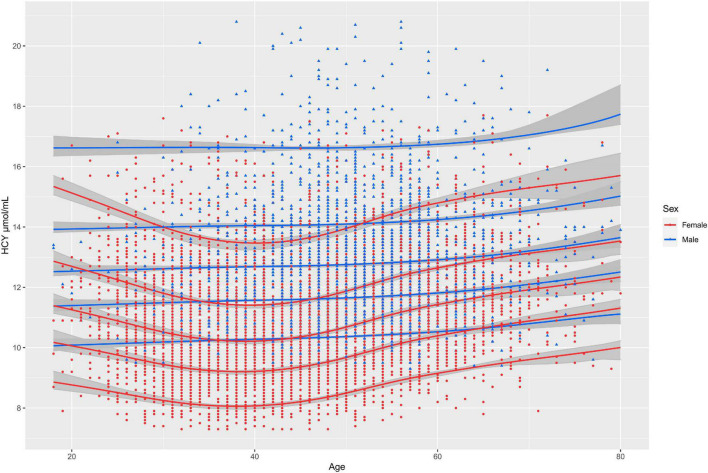
The solid lines from top to bottom represent the 95th, 75th, 50th, and 25th, 5th quantiles of concentration of Hcy, respectively; The gray shadows represent 95% confidence intervals for each percentile curve.

SBC of aging model of Hcy for female and male is 20609.71 and 9686.174, respectively. Aging model shows that the upper limit of the RI (P95) for Hcy is higher in males than in females at all ages. Models of females suggest that Hcy’ levels decline slightly with age beginning at age of 18 and begin to rise approximately after the age of 40. The Sex-specific aging model of Hcy for males showed that the upper limits of the RI of Hcy increased with age (Figure 3). FOR value for female and male model validation is 6.56 and 6.33%, which are both less than 10%.

## Discussion

Hyperhomocysteinemia has been demonstrated to associate with many diseases including cardiovascular disease, assessing the RIs for Hcy is a critical step for the correct interpretation of test results in all clinical laboratories. In the study, we not only analyzed the effects of sex and age on the levels of Hcy by using the variance components model, and five data mining algorithms were applied to establish the total and sex specific RIs of Hcy using real-world big data. Furthermore, we used GAMLSS algorithms to derive a sex-specific and continuous RI model of Hcy. This study not only provided a more personalized tool for clinicians to use Hcy, but also provides ideas and methodological reference for other clinical laboratories to establish RIs using real-world big data.

Our study population was derived from the physical examination population, since the characteristics and distribution of the physical examination population were more similar to the community population (14), and the results were also more representative relative to the outpatient and inpatient populations. Furthermore, we employed Tukey method to flag and remove potential outliers in subgroups to prevent interference of outliers in the establishment of RIs and the modeling process.

In the calculation of SDR, this study first used multiple linear regression to calculate the standardized regression coefficient of sex and age, which can be utilized to judge the influence of sex and age on Hcy concentration. The standardized regression coefficient of sex is larger than that of age. Therefore, when building the variance component model, sex was placed in the first layer of model independent variables, and age was placed in the second layer. According to the variance component model, sex’s SDR is greater than 0.375 while age is less than 0.375 (15, 16), indicating that the RI of Hcy should be divided according to sex. Previous (17, 18) studies only used multi-factor or single-factor methods to compare the test results between subgroups and judged whether the RI needed to be divided by whether the *P*-value was less than 0.05 or 0.01. This method is often unreliable in big data analysis, because the difference test is affected by sample size, and even minor differences between subgroups will be detected in a large sample size. Therefore, we recommend the method of SDR combined with the experience of clinicians to determine whether the RI needs to be partitioned.

In establishment of RIs for Hcy, the upper limits of the RIs established by five algorithms were higher in males than females. The sex differences are consistent with the previous study, which could be explained by differences in muscle mass, hormones, and vitamin status between females and males (12). Currently, the RIs of Hcy for most clinical laboratories are not partitioned by sex, which may lead to many males being wrongly identified as having elevated levels of Hcy, causing unnecessary panic and medical interventions. This is also wrongly identifying females as not having elevated homocysteine. In addition, although the principles of the five data mining algorithms are different, the results showed that the RIs established by the five algorithms had good consistency, which demonstrated the feasibility of using real-world big data to establish RIs of Hcy or analytes which have the similar distribution as Hcy.

There is a huge gap between the establishment of RIs and clinical practice. A possible solution is to embed a dynamic fitting model into laboratory testing platforms (19). The quantile curves which changed with age, such as 2.5th, 5th, 25th, 50th, 75th, 95th, and 97.5th percentiles could be easily obtained from the models. GAMLSS algorithm have been widely applied to implement and describe the complex age-dependent trends (20, 21). They also perform well at establishing continuous RIs for analytes (22). In the study, we found that RI of Hcy declined with age beginning at age of 18 and began to rise approximately after age of 40 for females and the tendency to rise with age is not clear in males. We also found in previous studies that level of other biomarkers like alkaline phosphatase fluctuated more with age in females than in males (19). We suspect that this may be due to the extreme fluctuation of female sex hormone levels with age. This increasing trend is similar to previous reports, except that the previous reports did not establish a continuous aging model and only plotted the curve of the mean of homocysteine with increase of age. To our knowledge, this is the first study to establish the complex age-dependent model for Hcy using real-world big data. The results showed that the Hcy level fluctuated with age in both sexes and the upper limit of the RI for Hcy was consistently higher in males than in females from the age of 18. Doctors could judge whether the individual’s result of Hcy is abnormal according to individuals’ age and sex. Moreover, the patient’s results can be plotted on a figure of the model so that the position of the patient’s results in the distribution of Hcy in the corresponding sex and age can be seen. In establishment of models, model with minimum SBC was selected as the best model. In internal validation, the FOR value of both male and female models was less than 10%, indicating that the age specific continuous RIs calculated by the model was of good applicability.

The medically determined levels of homocysteine currently in clinical use do not derive from the upper limit of the reference interval. Instead, a meta-analysis (23) used the median homocysteine of males plus 5 as the medically determined level. Interestingly, the upper limit of the reference interval established in the study is surprisingly consistent with this result. Furthermore, Kweon et al. (4) established reference interval for homocysteine in Korean population aged 45–74 years, in which the upper limit of the homocysteine’ reference interval was 13.80 μmol/L for males and 10.19 μmol/L for females. The results are lower than ours. This may be explained by the fact that we included people over 74. The oldest age of the participants in their study was 74. Another Indian study (24) showed that the upper limits of reference intervals for male and female Hcy were 16.50 and 16.27 μmol/L. Although different statistical methods were adopted. The RI was relatively close to the results of our study.

There are several limitations to this study. Firstly, other factors besides age and sex, such as specific dietary habits, and genetic, may also affect Hcy levels. In this study, we could not obtain such information. However, this happens to be the advantage of using data mining algorithms to establish RIs, that is, we do not need to know the exact situation of the included individuals. Just be sure to data used to analyze that includes a large number of healthy individuals. Based on this premise, the data mining algorithm can separate the distribution of healthy people from the cosmopolitan distribution. In addition, to compensate for this limitation, folate, vitamin B12, Cr, TSH, ALT, AST, Urea, FT4, and FT4 results was retrieved from the database to ensure that these analyts’ levels of the individuals used to establish the reference interval were normal. In addition, we used five algorithms to establish the RI, and the results were consistent. This also demonstrated the reliability of our results. Secondly, the model lacks external validation. In the future multi-center big data research, we will use the homogeneous data from other centers for external validation of the model. Thirdly, RIs defined this way not necessarily enable defining cardiovascular risk levels. The RI only represents the distribution range of Hcy in healthy individuals, and whether it has clear predictive value needs further study in the future.

## Conclusion

The RI of Hcy needs to be partitioned by sex and the upper limits of RI for Hcy was higher in males than females. The RIs established by the five data mining algorithms showed good consistency. The dynamic sex and age-specific model of Hcy showed the pattern of Hcy concentration with age and provide more personalized tools for clinical decisions.

## Data Availability Statement

The data analyzed in this study is subject to the following licenses/restrictions: The original contributions presented in the study are included in the article, further inquiries can be directed to the corresponding author. Requests to access these datasets should be directed to corresponding author.

## Author Contributions

CM, XC, and LQ designed the study. CM analyzed the data. CM, LH, LL, and XW wrote this manuscript. LH, YY, and LX performed detection of the analytes. All authors reviewed the manuscript.

## Conflict of Interest

The authors declare that the research was conducted in the absence of any commercial or financial relationships that could be construed as a potential conflict of interest.

## Publisher’s Note

All claims expressed in this article are solely those of the authors and do not necessarily represent those of their affiliated organizations, or those of the publisher, the editors and the reviewers. Any product that may be evaluated in this article, or claim that may be made by its manufacturer, is not guaranteed or endorsed by the publisher.
