# Supplementary material for: Establishment of Reference Interval and Aging Model of Homocysteine Using Real-World Data
Source: Front Cardiovasc Med. 2022 Mar 30;9:846685. doi: 10.3389/fcvm.2022.846685 (PMC9005842; doi:10.3389/fcvm.2022.846685)
Supplement: Supplementary file 1 [file Table_1.DOCX]

**Supplemental Table 1 SBC and FOR value of Ageing Model for Hcy by sex**

|  | **Group** | **GAMLSS** | |
| --- | --- | --- | --- |
|  |  | **FOR_U_** | **SBC** |
| **HCY** | **Female** | 6.56% | 20609.71 |
|  | **Male** | 6.33% | 9686.174 |

This table is the result of goodness of fit and internal validation of the model. A FOR value of less than 10% is acceptable.
